# The Nature of Alloys

**Published:** 1909-09-15

**Authors:** M. L. Ward


### THE NATURE OF ALLOYS.

BY M. L. WARD, D.D.SC.

Lecture to the Senior Dental Class, 1909.

At the last hour we had finished the subject of tin foil and begun the subject of amalgam. We defined the term alloy, as well as the term amalgam.

We called your attention to the fact that mercury dissolved all the metals except one or two making it possible to form a great number of alloys. We gave you an idea of the countless number of combinations thus possible as stated by Roberts—Austen to run up well into the thousands. The affinities between mercury and other metals was spoken of referring especially to the fact that mercury was removed from all its alloys by heat and most of them by pressure. Your attention was next directed to the different agencies by which the union of metals was affected. Three were mentioned, the first, and one usually employed, being heat, the second pressure and absence of heat entirely, and the third direct contact without heat or pressure. The statements of Matthiessen that it is probable that the condition of an alloy of two metals in the liquid state is either "a solution of one metal in another"—"a chemical combination"—"a mechanical mixture"—"a solution or mixture of two or all of the above;" and that a similar condition may exist in a solid state was brought to your attention with special reference to the dissolving of each of the constituents of dental amalgam alloys. Some of the peculiarities of chemical compounds, solutions and mechanical mixtures were discussed under the head of "Nature of Alloys".

It has been stated that it is pretty nearly a general rule that all metals which unite with oxygen to form only bases have a strong tendency to unite with metals some of whose oxides possess an acid character; and those which are allied in regard to basicity or acidity, have little affinity for each other.

Sodium and potassium afford a good example of allied materials, having practically no affinity for each other. They are miscible in various proportions, though they exhibit little or no tendency to unite in definite quantities. The same is true of antimony and tin. On the other hand copper and tin form stable alloys. There are some instances as in the case of lead and zinc where only very unequal portions can be made to unite, the main bulk of the metals separating on cooling. In most cases of mixed metals there is not the total and complete alteration of properties which is the distinctive feature of chemical action between a metal and a non-metal.

That you may grasp thoroughly the subject of alloys I want to refer frequently and with much emphasis to the fact that alloys *still possess the metallic character*, though a considerable evolution of heat is evinced, the melting points are lowered, the mean density is increased and the color and other physical properties considerably modified. The identity of each constituent can often times be traced through to the finished alloy. This, you will observe, is somewhat different from the greater part of the chemistry you have been taught. In the courses you have had your teachers have selected largely characteristic chemical reactions in which one element or compound has entered, with considerable energy, into combination with other elements or compounds to form a new substance with new and characteristic properties. Their object was largely to teach you the nature of bases, acids and salts and give you a working knowledge of affinities and energies. With our working knowledge of these phenomena I ask you to imagine that two or more metallic substances may unite, often with feeble affinities, to form a chemical compound, or they may form a simple mechanical mixtures of all three, though in anyinstance the resulting product is *metallic in nature.* It is not a new substance with new properties. Most of the metals with which we have to deal are capable to some extent of existing in a state of chemical combination with each other, but, as a general rule, they are united by feeble affinities and almost always are of a decidedly *metallic nature.*

It is probable that the metals with which we have to work do unite in definite proportions and form new compounds but it is difficult to obtain these compounds in a separate condition, since they dissolve in all proportions in the melted metals, and do not generally differ so widely in their melting points from the metals they may be mixed with, as to be separated by crystallization in a definite condition. If we are to get new compounds formed with loss of identity of the elements entering into it there must be more energetic union than we have when most of the metals unite. When copper and tin unite there is quite a change in color, the resulting product is brittle and otherwise differs from either the copper or tin. The product is generally represented by the formula Sn Cu2 and is a good example of a chemical combination but even here with what we might call a characteristic reaction the product is decidedly *metallic in nature* and is quite like either constituent. Such is nearly the case with mercury and silver which exist in the native state crystallized together in the proportions of one combining weight of silver to two or three of mercury.

When zinc and tin are put together or put into either of the above combinations that phenomena so allied to the action of acid on base seems to dissappear somewhat. The same is true with many of the combinations of which mercury is a part. It often unites with a single metal to give a product differing somewhat from either constituent, but when there are three or four metals to unite with it, the effect of combining substances which have allied properties again manifests itself. The question might well be asked here why alloys in the native state cannot be found and used for our work. Oftentimes alloys exist in the native state which quite meet our demands, but their quality can seldom be relied upon. It therefore becomes necessary to subject such products to preparation and purification. The work thus involved is equal to preparing artificial alloys. The scientific world has about abandoned hope of finding natural products which meet the requirements of the metallurgical world and are devoting their energies to the preparation of artificial alloys. We can hardly overestimate the value of these products when we consider that outside of one or two places in dentistry scarcely a single metal is used unalloyed.

That these bodies remain metallic in nature and do not lose their identity completely is of inestimable value to us not only in dentistry but in all lines of industries. Scarcely do we find a metal that possesses properties such as to render it suitable to be employed alone by the manufacturer. Even among the metals which *can* be used independently it is often found expedient to add portions of other metals to improve the color, hardness, plasticity, volumetric change, conductivity, etc. It is here that we really appreciate the value of having these products remain metallic in nature and not lose their identity completely when another metal is added to them. If a certain combination such as the coin silver and mercury, one of the first dental amalgams, were to lose most of its desirable properties as soon as some other metal were added we would not be able to use it at all. What I want to impress you with is the fact that the coin silver and mercury when mixed into an amalgam has its properties only slightly changed by the addition of small quantities of other metals. The original properties remain, but, slightly modified by the newly added metal. There are some exceptions to this but it is so generally true that we are almost always able to find some metal which will change some particular property that we want changed without changing other properties completely. The purposes for which the metals are alloyed are as various as the uses of the metals themselves but as a rule the combination is employed to harden, render more fusible, alter the color, lessen volumetric change, decrease conductivity, change the plasticity, or reduce the cost of production. The well known class of alloys termed "Brass" furnishes a good illustration of the effect of alloying in producing different shades of color. Brass is composed of copper and zinc in varying proportions, the color depending to a great extent on the quantity of copper present. When copper predominates the color is yellow or reddish; when the two metals exist in equal proportions the color is still yellow; beyond this, when the zinc is in excess, the color gets white or bluish-white resembling impure zinc. Some metals like copper are too tough to be turned on a lathe or cut with a file but when alloyed with tin or zinc can be readily turned, filed or rolled with considerable facility. In some cases tensile strength is greatly increased by the addition of another metal even in very small proportions, the various bronzes being examples. On the other hand the addition of a second metal may be a source of weakness as in the case of adding antimony to lead, and tin to silver and copper in dental amalgam alloys.

One might be lead to consider that the alloying of two malleable metals would produce a very malleable alloy and, while in many cases this is undoubtedly so there are others in which the opposite is the fact. Lead and gold are quite malleable but when even when a small quantity is added to gold the product is brittle and weak. The specific gravity of an alloy nearly always differs from the mean specific gravities of the constituents, sometimes being greater and sometimes less. When the density is increased it shows that contraction has taken place and chemical combination has probably taken place between the components. This is a phenomena of vital importance to the dentist and one which so far has not been totally eliminated from amalgam alloys. When mercury unites with silver or zinc the specific gravity is decreased and we say there is expansion, while if mercury and tin are united the specific gravity is increased and we observe the phenomena of shrinkage. When we come to study dental amalgam alloys and their application to dentistry we shall be convinced forcibly that of all the phenomena spoken of under the head of "Nature of Alloys" the last one mentioned interests us most. From the time amalgam alloys were first mentioned early in the 19th century till Black's work in 1895 shrinkage was the phenomena hardest to eliminate while from the time of Black's work in 1895 until the present time expansion has been the most undesirable feature.

As a rule it is more difficult to alloy three or four metals than two metals, especially when the components differ widely in fusibility, unless the combination forms a true chemical compound. This is particu'arly true with most of our present alloys which are composed of four metals exclusive of the solvent, mercury. When this is considered together with the great difficulties connected with the separating out of the constituents according to their specific gravities on cooling, we should not be surprised when we find later that Black thought no given formula could be used to bring about a product with no shrinkage and a minimum amount of expansion. With this preliminary consideration of the nature of alloys we will now begin a somewhat detailed study of the subject commonly known among dentists as amalgams, but properly known as dental amalgam alloys. This class of compounds is usually made up of four metals viz.—silver, tin, copper and zinc, which after being alloyed have been turned into filings and mixed with a fifth metal, mercury. When the first four mentioned metals are mixed with the mercury and allowed to stand, the mass becomes hard and we say it has set. The phenomena of setting is probably due to the formation of crystals, the greater part of which are formed from the action of silver on mercury. These crystals whether formed from silver and mercury or from some other metal and mercury may bind together the mass the interstices of which may be undissolved particles of alloy or solutions of some of the metals in mercury. The thing of importance to us now is that mercury dissolves the alloy formed from the first four metals and unites with a portion or all of it to form crystals, these crystals in turn binding the mass together. This as you will observe is one of the most complex alloys known and involves many of the phenomena we have already called your attention to under the head of "Nature of Alloys."
So far as is known at present no definite method of combining the first four metals has been reached though the students of Black claim to have mastered the art. They claim that owing to irregularities in the behavior of different batches of metal no "set formula" can be used, though they fail to set forth any order of melting the constituents in the plan of alloying these constituents though at present the work is not ready for publication.

When you consider how little is really known of the structure of these bodies before they are alloyed with mercury together with the fact that the difficulty is further multiplied by the addition of mercury, I think you will see that we cannot say more than that mercury dissolves the alloy of four metals and forms crystals which set the mass. We cannot say that a certain number of combining weights of mercury unite with a certain number combining weights of the alloy, and the chances are that we never will be able to do so if dental amalgams continue to be made of five metals. You have probably some idea of the great number of possibilities when an alloy of five metals is formed, but when you consider the number of metals used here which have allied properties the magnitude of the mystery must be apparent. There are still made and in the market alloys composed of two and three metals which are combined with mercury to form an amalgam. When two metals are used to form the alloy they are usually silver and tin though some of the older alloys of silver and copper are still used. If the physical characteristics of these ternary and quarternary amalgams were desirable enough to warrant a more generous use of them, undoubtedly enough interest would be taken in them to discover their structure, but it is a hopeless task to determine the structure of an amalgam composed of five metals whose properties are so similar as the ones now used for this purpose. For the present let us content ourselves with the fact that mercury dissolves one or more metals and the mass when let alone sets. During the setting process a great many of the phenomena already spoken of may take place. There may be a change in density and this of course may cause shrinkage or expansion. There may be a change in strength, change in color, or a number of the phenomena we have called your attention to. Many fail to observe that, from a structural standpoint, there are a number of possibilities when mercury dissolves an alloy of two or more metals to form an amalgam, while the same number fail to realize that almost all the laws of alloying apply to the union of mercury with dental alloys. We will now examine one of Black's charts which shows some of these phenomena when silver and tin are alloyed and this alloy subsequently alloyed with mercury to form a ternary amalgam.

BLACK'S CHART AS PER COSMOS P. 982.

FORMULAE.

| Silver | Tin | How Prepared | Per Cent. of Mercury | Shrinkage | Expansion | Flow | Crushing Stress |
|---|---|---|---|---|---|---|---|
| 40 | 60 | Fresh-cut | 45.78 | 6 | 7 | 40.15 | 178 |
| 40 | 60 | Annealing | 34.14 | 9 | 3 | 44.60 | 186 |
| 45 | 55 | Fresh-cut | 49.52 | 4 | 8 | 25.46 | 188 |
| 45 | 55 | Annealed | 32.13 | 11 | 1 | 28.57 | 222 |
| 50 | 50 | Fresh-cut | 51.18 | 2 | 2 | 22.16 | 232 |
| 50 | 50 | Annealed | 37.58 | 17 | 1 | 21.03 | 245 |
| 55 | 45 | Fresh-cut | 51.62 | 2 | 2 | 19.66 | 245 |
| 55 | 45 | Annealed | 40.11 | 18 | 0 | 17.53 | 276 |
| 60 | 40 | Fresh-cut | 52.00 | 1 | 0 | 9.06 | 239 |
| 60 | 40 | Annealed | 39.80 | 17 | 0 | 14.10 | 297 |
| 65 | 35 | Fresh-cut | 52.00 | 0 | 1 | 3.67 | 290 |
| 65 | 35 | Annealed | 33.00 | 10 | 0 | 5.00 | 335 |
| 70 | 30 | Fresh-cut | 55.00 | 0 | 14 | 3.45 | 316 |
| 70 | 30 | Annealed | 40.00 | 7 | 0 | 4.67 | 375 |
| 72.5 | 27.5 | Fresh-cut | 55.00 | 0 | 42 | 3.94 | 275 |
| 72.5 | 27.5 | Annealed | 45.00 | 3 | 0 | 376 | 362 |
| 75 | 25 | Fresh-cut | 55.00 | 0 | 60 | 5.64 | 300 |
| 75 | 25 | Annealed | 50.00 | 0 | 6 | 5.40 | 300 |

From this chart you will observe that Black found that when any one of the nine different proportions of silver and tin were alloyed with mercury there was change in volume. In some cases the change was merely an increase in volume while in others it was simply a decrease. In many cases, especially when 40 per cent. to 55 per cent. of silver was used, there was first a decrease in volume and then an increase. You will also observe that every one of the different combinations were annealed after being cut and then alloyed with mercury. The difference between the two manifests itself first by a smaller percentage of mercury being required for the alloy which has been annealed, and second by a less amount of expansion and greater amount of shrinkage taking place with the alloy which has been annealed. By observing closely the effect of the annealing you will also note that the change produced is less with the smallest amount of silver used and greatest with the highest amount used. The change, whlle it appears to be a gradual one depends upon the amount of silver, seems to dissappear somewhat when more than 75 per cent. of silver is used. In the column marked "flow" some interesting phenomena appear. A casual observation will show that the resistance to flow is increased with the percentage of silver, though the part of it which will interest us most, when we leave the subject, "Nature of Alloys," at the next hour and begin a close and critical study of dental alloys, is the increase which appears so abruptly at 65 per cent of silver.

